# Urinary Peptides Associated Closely with Gestational Diabetes Mellitus

**DOI:** 10.1155/2020/8880034

**Published:** 2020-08-20

**Authors:** Zhiying Hu, Yaping Tian, Jia Li, Mei Hu, Man Zhang

**Affiliations:** ^1^Clinical Laboratory Medicine, Beijing Shijitan Hospital, Capital Medical University, Beijing 100038, China; ^2^Beijing Key Laboratory of Urinary Cellular Molecular Diagnostics, Beijing 100038, China; ^3^Laboratory of Translational Medicine, The First Medical Center of Chinese PLA General Hospital, Beijing 100853, China

## Abstract

Gestational diabetes mellitus (GDM) is a common disease of pregnant women, which has a higher incidence in recent years. The purpose of this study is to explore urinary biomarkers that could predict and monitor gestational diabetes mellitus (GDM). Urine samples from 30 normal pregnant women and 78 GDM patients were collected and purified by weak cationic exchange magnetic beads (MB-WCX), then analyzed by matrix-assisted laser desorption ionization time of flight mass spectrometry (MALDI-TOF-MS). The urinary peptide signatures of the two groups were compared by BioExplorer software. The potential ability of the differently expressed peptides to distinguish GDM patients from normal pregnant women was evaluated by receiver operating characteristic (ROC) analysis. At last, the differently expressed peptides were identified by liquid chromatography tandem mass spectrometry (LC-MS). There were four differently expressed peptides (*m*/*z* 1000.5, 1117.5, 1142.9, and 2022.9) between two groups, which were identified as fragments of urinary albumin, *α*2-macroglobulin, human hemopexin, and *α*1-microglobulin, respectively. The diagnostic efficacy of *m*/*z* 1142.9 was better than the other peptides. The area under the curve (AUC) of the *m*/*z* 1142.9 was 0.690 (95% CI: 0.583-0.796). The discovery of urinary polypeptides provides the possibility for the early prediction of GDM and the monitoring of glucose metabolism in GDM patients by a noninvasive method.

## 1. Introduction

Gestational diabetes mellitus (GDM) can increase the rate of miscarriage, lead to fetal growth restriction, fetal malformation, macrosomia, neonatal respiratory distress syndrome, neonatal hypoglycemia, and other adverse prognoses, and significantly increase the probability of type 2 diabetes in mothers and offspring in the long term [1–5]. Several studies show that GDM treatment can reduce the incidence of adverse pregnancy outcomes [6–8]. In order to monitor the glucose metabolism of GDM patients, the fasting plasma glucose (FPG) and glycosylated hemoglobin are currently used in the clinic. FPG detection can realize the real-time monitoring of glucose metabolism, but it needs repeatedly invasive blood collection operations by nurses. The traumatic operation leads to the poor compliance of GDM patients. Although glycosylated hemoglobin can effectively reflect the blood glucose level of GDM patients in the past 1-2 months, irreversible organ damage to pregnant women and fetuses may have occurred.

In recent years, urinary proteomics has developed rapidly. As the end metabolite, urine has many advantages, such as convenient collecting, completely noninvasive, accumulating more protein types, and reflecting more body pathological changes [9]. As the Beijing Key Laboratory of Urinary Cellular Molecular Diagnostics, the research on small molecular polypeptides in urine of patients with type 2 diabetes has achieved preliminary results [10]. Through the study of urine polypeptides combined with plasma glucose in GDM patients, we hope to provide objective test indexes for the primary screening and auxiliary diagnosis of GDM. The discovery of biomarkers in urine also lays a foundation for the study of the pathological mechanism of GDM and provides a possibility for the prediction and dynamic monitoring of glucose metabolism in patients.

## 2. Materials and Methods

### 2.1. Study Population

Firstly, the ethics committee of Beijing Shijitan Hospital, Capital Medical University, approved the research project (Research Ethics No. (27) 2018). Secondly, the subjects were all female, aged 24-42 years; 78 subjects were GDM patients in our hospital from April 2018 to August 2019 (GDM group); 30 subjects were normal pregnant women who completed routine obstetric examination in our hospital in the same period (N group). The inclusion criteria are the following: (1) The clinical data of GDM patients and normal pregnant women from 8 weeks to 42 days after delivery were complete. (2) One-step oral glucose tolerance test (OGTT) with 75 g glucose was performed at 24-28 weeks of gestation. (3) The diagnosis of GDM meets the diabetes diagnosis standard of the American Diabetes Association (ADA) in 2011. The exclusion criteria are the following: (1) GDM subjects with impaired glucose tolerance in the past; (2) all subjects with acute or chronic infections, tumors, and cardiovascular diseases; (3) subjects with severe liver and kidney dysfunction; and (4) GDM subjects with complications such as anemia, abnormal thyroid function (hyperthyroidism and hypothyroidism), pregnancy-induced hypertension syndrome (PIH), preeclampsia, and so on.

78 GDM patients were divided into GM1 (FPG ≤ 4.50 mmol/l (81 mg/dl)) and GM2 (FPG > 4.50 mmol/l (81 mg/dl)) groups according to the fasting plasma glucose (FPG) level in the OGTT. The clinical characteristics of all subjects are compared as shown in Table 1.

### 2.2. Urine Sample Collection Preparation

All subjects were informed to refrain from heavy physical activity the day before urine collection. The second void morning urine samples were collected. The urine samples of all subjects had no hematuresis or ketosis. The methods including urine sample pretreatment and temporary storage, fractionation of urinary peptides using weak cationic-exchange magnetic beads (Bioyong Technologies Inc, Beijing, China), MALDI-TOF-MS AnchorChip spotting (Bioyong Technologies Inc, Beijing, China), and data acquisition were all performed as previously developed by Hu et al. [11].

### 2.3. Statistical Analyses

Descriptive patient characteristics were displayed as the mean ± SD unless otherwise indicated, and calculations were performed using SPSS 17.0. The FPG results measured by OGTT were collected from GDM patients who have no food intake for more than 8 hours in their second trimester. The peak area was used as quantitative standardization. The comparison of the peak area between two groups was performed by *t*-tests (normal distributed data) or Wilcoxon test (abnormal distributed data) using BioExplorer software. Two-tailed *P* values < 0.05 were considered significant in all statistical comparisons. ROC curve analysis and AUC calculations were performed directly with SPSS 17.0 software to determine diagnostic efficacy of each single marker. A binary logistic regression model was established to evaluate the multivariate diagnostic value.

### 2.4. Peptide Sequence

The 20 *μ*l prepared sample of each subject was injected once and identified by LC-MS. The HPLC system EASY-nLC1000 (Thermo Fisher) was used for separation. The liquid phase A was 0.1% acetonitrile formate solution (2% acetonitrile), and the liquid phase B was 0.1% acetonitrile formate solution (98% acetonitrile). The C18 chromatographic column (Thermo Fisher) was equilibrated with 100% A solution. The flow rate was 200 nl/min. Gradient elution profile was as follows: 2% B-6% B-22% B-32% B-100% B-100 %B in 60 minutes. The samples were separated by capillary high-performance liquid chromatography and analyzed by a Q Exactive plus spectrometer (Thermo Scientific).

### 2.5. Bioinformatics and Identification of Urine Biomarkers

The spectra were analyzed with Peaks8.5 (Bioinformatics Solutions Inc.), and the resulting mass lists were matched against the IPI Human database (v3.45) using Sequest search. Parameters were set as follows: Delton ≥ 0.1; charge2+, Xcorr2.0; charge3+, Xcorr2.5; peptide probability ≤ 1*e* − 003; parent ion mass tolerance: 10 ppm; fragment ion mass tolerance: 0.02 Da; enzyme: no enzyme; variable modification: oxidation of methionine.

## 3. Results

### 3.1. Urinary Peptidome Profiling

**Figure 1:**
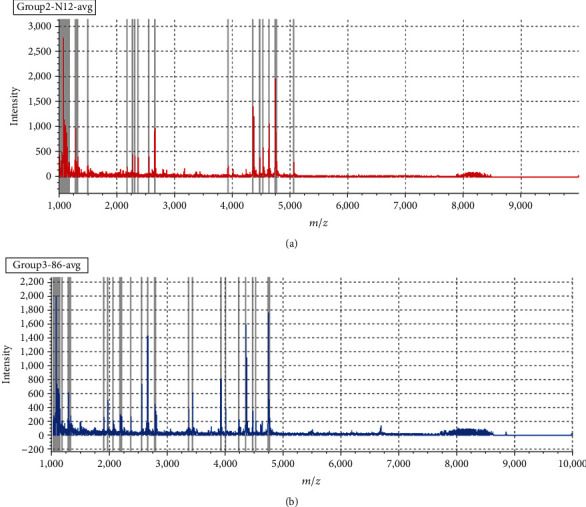
Typical urinary sample mass spectra from MALDI-TOF-MS after being purified by MB-WCX: (a) one sample of a normal pregnant woman; (b) one sample of a GDM patient.

Urine samples from 108 volunteers were purified by MB-WCX. After analysis of MALDI-TOF-MS, typical WCX spectra are shown in Figure 1.

### 3.2. Statistical Analysis between N Group and GDM Group

**Figure 2:**
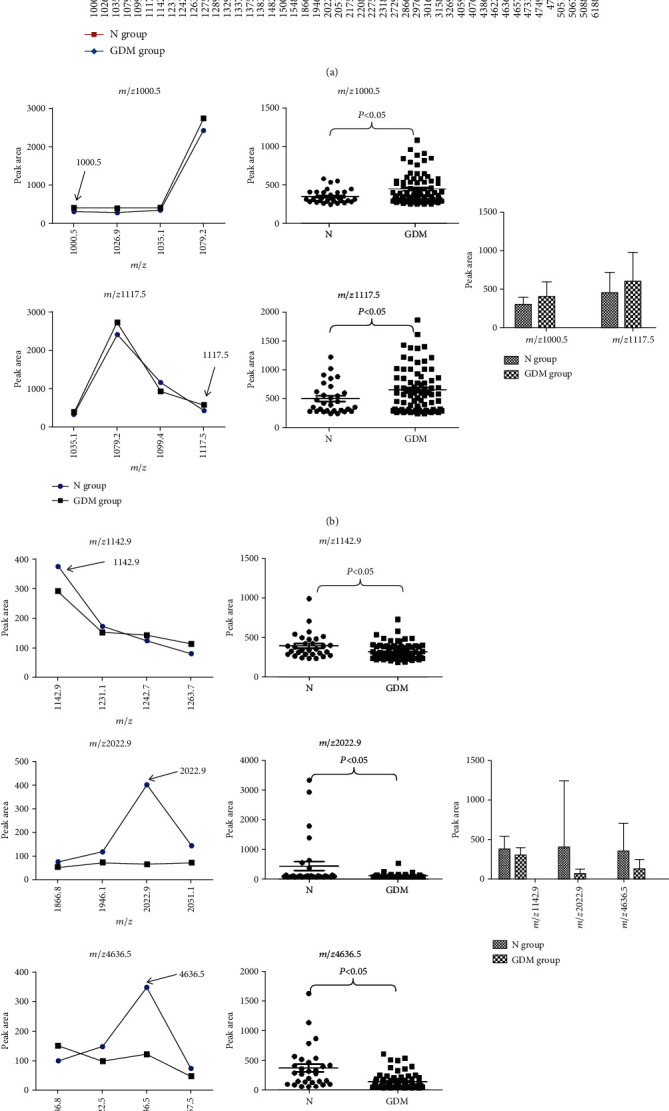
Urine polypeptides in the N group and the GDM group were analyzed. The samples in the N and GDM groups were urine samples of 30 normal pregnant women and 78 GDM patients, respectively. (a) The average peak area distributions of all polypeptide peaks were measured in two groups. Five urine polypeptides which were indicated by the arrow were statistically significant in the comparative analysis of two groups (*P* < 0.05). (b) In the comparative analysis of five polypeptides between the N and GDM groups, there were significant differences in the *m*/*z* 1000.5 and 1117.5 (left, *P* < 0.05). The distributions of two polypeptides in two groups are shown (middle). The peak value of two polypeptides in the GDM group was higher than that in the N group (right, *P* < 0.05). (c) There were significant differences in the *m*/*z* 1142.9, 2022.9, and 4636.5 (left, *P* < 0.05). The distributions of three polypeptides in two groups are shown (middle). The peak value of three polypeptides in the GDM group was lower than that in the N group (right, *P* < 0.05).

Using BioExplorer software, 172 distinguishable peaks were detected within the 1,000 to 10,000 mass charge ratio (*m*/*z*) range; 46 peaks have differential expression and statistical significance (*P* < 0.05) between two groups. We picked eight relatively higher peaks (peak area > 300) for further analysis. The peptides with a mass charge ratio of 1078.6, 1289.6, and 1501.8 had been published [11], and the mass charge ratio of the other five urine peptides was 1000.5, 1117.5, 1142.9, 2022.9, and 4636.5, respectively (Figure 2(a)). Compared to the N group, *m*/*z* 1000.5 and 1117.5 were upregulated (Figure 2(b)) and *m*/*z* 1142.9, 2022.9, and 4636.5 were downregulated in the GDM group (Figure 2(c)).

### 3.3. Statistical Analysis between GM1 Group and GM2 Group

**Figure 3:**
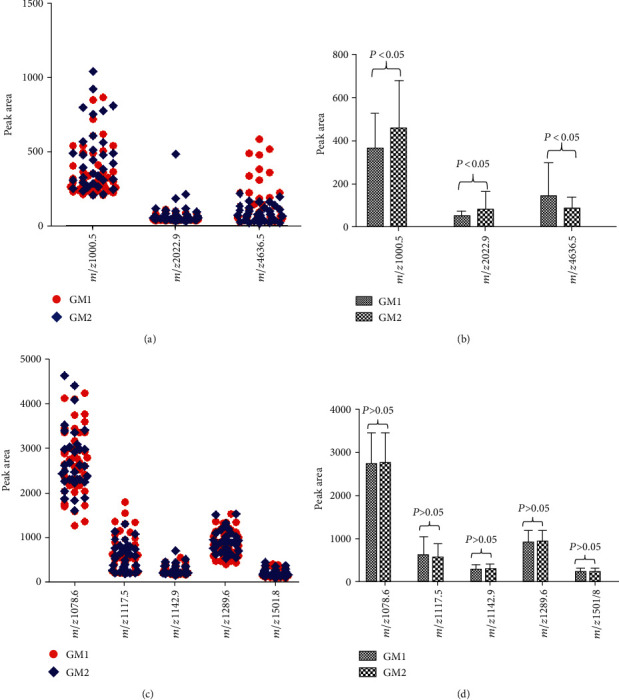
Comparison of the difference of urine peptides between the GM1 (*n* = 45) and GM2 (*n* = 33) groups. (a) The distribution of molecules with *m*/*z* 1000.5, 2022.9, and 4636.5 in two groups is shown. (b) There were significant differences between the GM1 and GM2 groups in three molecules with *m*/*z* 1000.5, 2022.9, and 4636.5 (*P* < 0.05). (c) The distribution of molecules with *m*/*z* 1078.6, 1117.5, 1142.9, 1289.6, and 1501.8 in two groups is shown. (d) There was no significant difference between the GM1 and GM2 groups in three molecules with *m*/*z* 1078.6, 1117.5, 1142.9, 1289.6, and 1501.8 (*P* > 0.05).

The differences of urine polypeptides between the GM1 and GM2 groups with *m*/*z* 1000.5, 1078.6, 1117.5, 1142.9, 1289.6, 1501.8, 2022.9, and 4636.5 were analyzed by BioExplorer software. The distribution of *m*/*z* 1000.5, 2022.9, and 4636.5 molecules in two groups was shown in Figure 3(a), and the differences between the two groups were statistically significant (*P* < 0.05, Figure 3(b)). Compared with the GM1 group, *m*/*z* 1000.5 and 2022.9 were upregulated and *m*/*z* 4636.5 was downregulated. The distribution of urine polypeptides of *m*/*z* 1078.6, 1117.5, 1142.9, 1289.6, and 1501.8 in two groups was shown in Figure 3(c), and there was no significant difference between the two groups (*P* > 0.05, Figure 3(d)).

### 3.4. Trend Analysis

**Figure 4:**
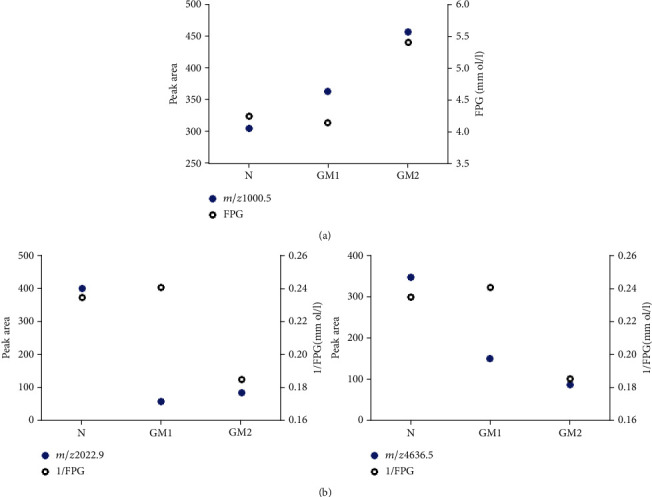
Trend analysis of differential polypeptide peaks and FPG. (a) Trends of FPG and the molecule with *m*/*z* 1000.5 in the N group (*n* = 30), GM1 group (*n* = 45), and GM2 group (*n* = 33). (b) Trends of 1/FPG and the molecules with *m*/*z* 2022.0 and 4636.5 in three groups.

With the increase of FPG in GDM patients, the expression of urine polypeptide with *m*/*z* 1000.5 increased; the expression of urine polypeptides with *m*/*z* 2022.9 and 4636.5 decreased. The trend between the molecule with *m*/*z* 1000.5 and FPG was better than that of the other two molecules (Figure 4).

### 3.5. ROC Analysis

**Figure 5:**
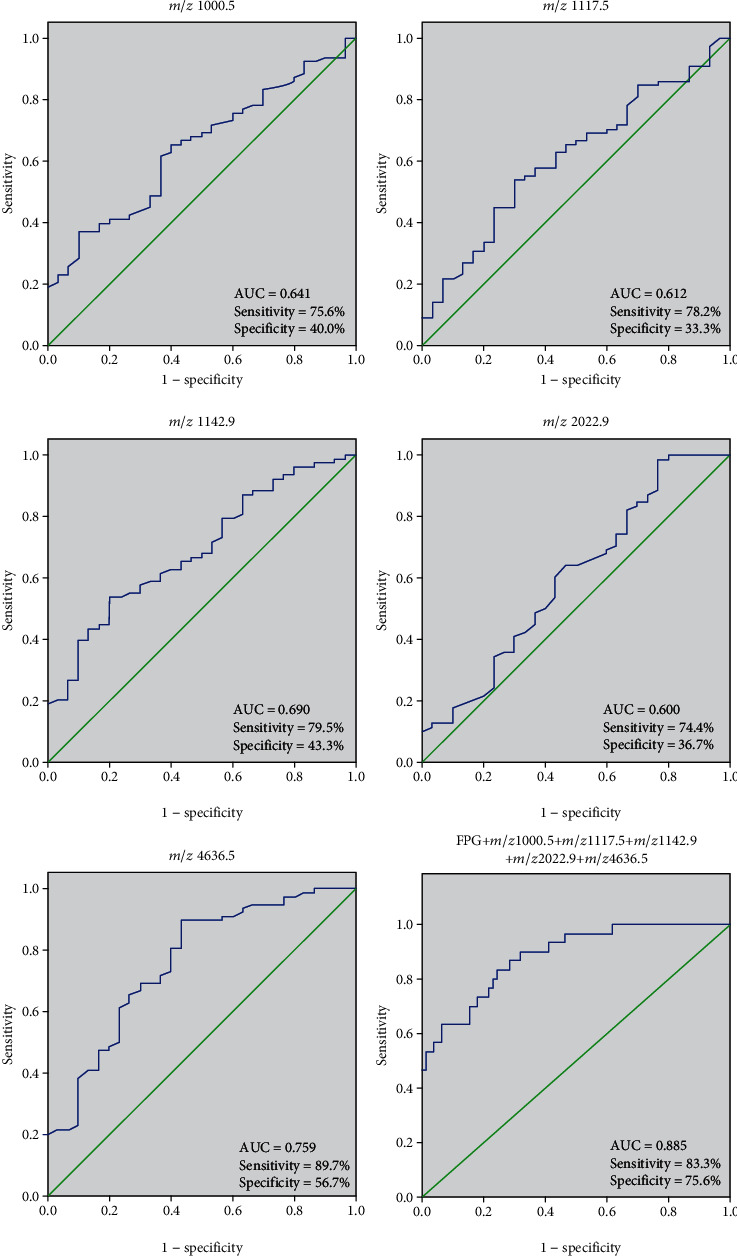
ROC analysis. The ROC curve and the AUC were established according to the peak value of urine polypeptides of GDM patients (*n* = 78) and normal pregnant women (*n* = 30) with *m*/*z* 1000.5, 1117.5, 1142.9, 2022.9, and 4636.5 and comprehensive index.

To evaluate the diagnostic efficacy of these peptides, the ROC analysis was performed to calculate the sensitivities, specificities, and accuracies at different cutoff points for differentiating GDM patients from normal pregnant women (Figure 5). In the ROC curves, the AUC of the peptides with *m*/*z* 1000.5, 1117.5, 1142.9, 2022.9, and 4636.5 were 0.641 (95% CI: 0.532-0.750), 0.612 (95% CI: 0.497-0.726), 0.690 (95% CI: 0.583-0.796), 0.600 (95% CI: 0.476-0.724), and 0.759 (95% CI: 0.655-0.863), respectively.

In the five indicators, polypeptide with *m*/*z* 4636.5 had the best diagnostic value for GDM, with a cutoff value of 235; its sensitivity and specificity were 89.7% and 56.7%, respectively. Multivariate logistic regression analyses including FPG and polypeptides with *m*/*z* 1000.5, 1117.5, 1142.9, 2022.9, and 4636.5 were used to evaluate their diagnostic values. The diagnostic formula was

(1) $$\begin{array}{c} Y=\text{logit}\left(P\right)=-5.393+1.710X_{\text{FPG}}+0.007X_{1000.5}+0.001X_{1117.5}-0.007X_{1142.9}-0.009X_{2022.9}-0.004X_{4636.5}. \end{array}$$

The AUC of multivariate logistic regression was 0.885 (95% CI: 0.817–0.952). At the cutoff value of 0.774, the sensitivity and specificity were 83.3% and 75.6%, respectively.

### 3.6. Identification of the GDM Potential Urinary Biomarkers

Four molecules with *m*/*z* 1000.5, 1117.5, 1142.9, and 2022.9 were analyzed by LC-MS, and their amino acid sequences were QTALVELVK, QTVSWAVTPK, DYFMPC (+57.02) PGR, and VVAQGVGIPEDSIFTM (+15.99) ADR. The corresponding names of four molecules were urine albumin (ALBU) precursor, alpha2-macroglobulin (A2MG) precursor, human hemopexin (HEMO) precursor, and alpha1-microglobulin (AMBP) precursor by database search. Unfortunately, the identification of the molecule with *m*/*z* 4636.5 failed. The detailed results are shown in Table 2.

## 4. Discussion

During pregnancy, progressive insulin resistance begins in the second trimester and develops further in the third trimester. Hormones and adipokines secrete by the placenta may be the cause of insulin resistance during pregnancy. Insulin sensitivity begins to decline gradually in the second trimester and becomes more serious in the third trimester [12]. In addition, the increase of estrogen, progesterone, and cortisol during pregnancy helps to destroy the glucose insulin balance [13]. In order to adapt to the insulin resistance and the reduction of sensitivity during pregnancy, insulin secretion increases. GDM occurs when the pancreas does not produce enough insulin to maintain metabolic pressure. GDM can cause a variety of obstetric complications such as hypertension, preeclampsia, premature rupture of membranes, and premature delivery [14–17]. Early prediction of GDM and monitoring of the patients' glucose metabolism level are very important for maternal and infant health.

Urinary proteome is the direction of disease diagnosis, treatment, monitoring, and prognosis research [9, 18], and its application fields include urogenital system and other system diseases [19–22]. Comparison of protein patterns in biological fluids between healthy individuals and patients with disease is increasingly being used both to discover biological markers of disease (biomarkers) and to identify biochemical processes important in disease pathogenesis [23]. As the Beijing Key Laboratory of Urinary Cellular Molecular Diagnostics, by analyzing the differences of urinary polypeptide peaks between GDM patients and normal pregnant women, we hope to fully tap the biomarkers of urinary polypeptides in GDM. We grouped GDM patients according to the FPG of OGTT and analyzed the correlation between different polypeptides and disease severity. Four peptides with significant difference were screened out and identified successfully. The protein names are urine albumin (ALBU), alpha2-macroglobulin (A2MG), human hemopexin (HEMO), and alpha1-microglobulin (AMBP).

The content of urine microalbumin (UmAlb) in the first trimester is less than 20 mg/24 h. As pregnancy continues, UmAlb may slightly increase, but it can remain in the normal range [24]. In the second and third trimesters, the sensitivity of insulin decreases and the demand for insulin increases. GDM patients cannot compensate for the physiological change; then, their plasma glucose rise. The disease results in hypoxia of tissues, increase of blood viscosity, vascular disease, increase of permeability of glomerular basement membrane, and glomerular damage mainly caused by microvascular disease [25]. Several studies show that the detection of UmAlb is a sensitive index for the diagnosis of early renal injury, and it is also the earliest clinical manifestation of diabetic renal microvascular damage [26–29]. GDM-related research points out that the UmAlb level of GDM patients is higher than that of normal pregnant women. Moreover, GDM patients who did not meet the standard of blood glucose control excreted more urine microalbumin. It is suggested that the increase of UmAlb excretion is one of the reasons for GDM progression [24]. In this study, the ratio of microalbumin to creatinine in urine of all subjects was less than 30 mg/g. However, through our urine proteomics study, we can detect the subtle change of UmAlb, and this change is enough to distinguish GDM patients from normal pregnant women. After grouping GDM patients according to FPG, the urinary polypeptide expression in the GM2 group was significantly higher than that in the GM1 group (*P* < 0.05). It indicated that the UmAlb expression increased with the increase of FPG in GDM patients.

Alpha2-macroglobulin (A2MG) is synthesized by hepatocyte and monocyte macrophage system, which is the largest protein in plasma. It is a major plasma protease inhibitor that also regulates the activity of a variety of bioactive peptides including interleukins and exerts a range of immunomodulatory effects [30]. In normal circumstances, A2MG cannot be filtered by the glomerulus, and its content in urine is very little. When the glomerular basement membrane is seriously damaged or blood components enter the urine, the A2MG level in urine increases [31]. According to diabetes researches, A2MG levels were significantly raised in the diabetes type I group. In the group of diabetes type II, A2MG levels are within the normal range. After division of diabetics according to the presence of diabetic complications, A2MG levels in patients with diabetic complications were significantly higher than in the group of diabetics without complications [32, 33]. The increase in plasma A2MG levels in diabetes may be a correlative measure to encounter the potential proteolytic challenge associated with diabetic microangiopathy, even very early in the course of the disease. A2MG may yet be one of the most specific markers of microvascular complications in diabetes than any other serum protein [30]. At present, there is no report about urine A2MG in GDM. This study showed that the expression of urine A2MG in GDM patients was increased compared with that of normal pregnant women, which was mainly related to glomerular microvascular lesions and increased basement membrane permeability. However, there was no significant difference between GM1 and GM2. Therefore, microexpression of urine A2MG of GDM patients may not be suitable as a marker of glomerular membrane damage.

Human hemopexin (HEMO) is a plasma beta-glycoprotein that specifically binds one heme with high affinity and transports it to hepatocytes for salvage of the iron [34]. Some researchers point out that hemopexin is upregulated in plasma from type 1 diabetes mellitus patients due to the effect of glucose-induced reactive oxygen species [35]. There is no report on the relationship between urinary HEMO and GDM. The increase of plasma in normal pregnancy was more than the increase of blood cells, resulting in the dilution of blood and relative anemia. This study found that the expression of urine HEMO in GDM patients was lower than that of normal pregnant women. We consider that although there is glucose-induced reactive oxygen species in GDM patients, the increase of plasma osmolality and blood volume is more obvious, so the final manifestation is the decrease of HEMO expression. The expression of HEMO in the GM2 group was higher than that in the GM1 group, but the difference was not significant.

Urinary AMBP provides a noninvasive and cheap diagnostic method for the diagnosis and monitoring of urinary tract diseases, which can detect renal tubular diseases of diabetic nephropathy in the early stage [36]. GDM-related studies indicated that the urinary AMBP in the GDM group was significantly higher than that in normal pregnant women and normal nonpregnant women. The combined detection of urinary microalbumin and AMBP can early detect the GDM nephropathy, which is a sensitive and effective index for early renal damage monitoring of GDM nephropathy [37]. In this study, compared with normal pregnant women, the urinary AMBP of GDM patients was significantly lower. When the renal tubular reabsorption function is normal, the excretion of urine AMBP will be less than that in normal pregnant women. However, the secretion of AMBP in the GM2 group was significantly higher than that in the GM1 group. It is suggested that with the increase of FPG, renal hypoxia and ischemia aggravated, resulting in glomerular filtration and renal tubular reabsorption dysfunction. With the further increase of blood volume in the third trimester, the secretion of AMBP in GDM patients was lower than that in the second trimester.

Through the study of urinary proteomics of GDM, we further explored the disease-related small urine polypeptides. Combined with FPG, the research results are expected to serve as the basis for the study of the urine glucose metabolism level detection kit. The study also provides early prediction, noninvasive diagnosis, treatment guidance, and prognosis information for GDM patients.

## Figures and Tables

**Table 1:** The clinical characteristics of all subjects (*X* ± *S*).

Parameters	N group (*n* = 30)	GDM group (*n* = 78)	GM1 group (*n* = 45)	GM2 group (*n* = 33)
Age (year)	31.83 ± 3.71	32.88 ± 4.21	32.38 ± 4.11	33.58 ± 4.32
Prepregnancy BMI	21.26 ± 2.52	23.35 ± 3.45	22.63 ± 3.61	24.32 ± 3.01
Average gestational age	39.54 ± 1.08	38.97 ± 1.95	39.32 ± 1.18	38.50 ± 2.61
Average number of pregnancies	1.90 ± 0.99	2.13 ± 1.21	2.02 ± 1.03	2.27 ± 1.42
Average number of births	1.40 ± 0.50	1.41 ± 0.55	1.38 ± 0.53	1.45 ± 0.56
Alb/Cr (mg/g)	<30	<30	<30	<30

Notes: Alb/Cr represents the ratio of microalbumin to creatinine in urine.

**Table 2:** Identification of the GDM potential urinary biomarkers.

*m*/*z*	Molecular weight	Amino sequence	Protein name
1000.5	999.6	QTALVELVK	Urine albumin precursor
1117.5	1115.6	QTVSWAVTPK	Alpha2-macroglobulin precursor
1142.9	1141.5	DYFMPC (+57.02) PGR	Human hemopexin precursor
2022.9	2020.0	VVAQGVGIPEDSIFTM (+15.99) ADR	Human alpha1-microglobulin precursor
4636.5	Identification failure		

## Data Availability

The necessary data of this manuscript has been shown in the article.
